# Advances in Nanomaterials for Injured Heart Repair

**DOI:** 10.3389/fbioe.2021.686684

**Published:** 2021-08-25

**Authors:** Jiacheng Guo, Zhenzhen Yang, Xu Wang, Yanyan Xu, Yongzheng Lu, Zhen Qin, Li Zhang, Jing Xu, Wei Wang, Jinying Zhang, Junnan Tang

**Affiliations:** ^1^ Department of Cardiology, the First Affiliated Hospital of Zhengzhou University, Zhengzhou, China; ^2^ Key Laboratory of Cardiac Injury and Repair of Henan Province, Zhengzhou, China; ^3^ Department of Oncology, the First Affiliated Hospital of Zhengzhou University, Zhengzhou, China; ^4^ Department of Medical Record Management, the First Affiliated Hospital of Zhengzhou University, Zhengzhou, China; ^5^ Department of Cardiac Surgery, the First Affiliated Hospital of Zhengzhou University, Zhengzhou, China; ^6^ Henan Medical Association, Zhengzhou, China

**Keywords:** nanomaterials, tissue engineering, therapeutic strategies, biomimetic, nano-gel, cardiac regeneration

## Abstract

Atherosclerotic cardiovascular disease (ASCVD) is one of the leading causes of mortality worldwide. Because of the limited regenerative capacity of adult myocardium to compensate for the loss of heart tissue after ischemic infarction, scientists have been exploring the possible mechanisms involved in the pathological process of ASCVD and searching for alternative means to regenerate infarcted cardiac tissue. Although numerous studies have pursued innovative solutions for reversing the pathological process of ASCVD and improving the effectiveness of delivering therapeutics, the translation of those advances into downstream clinical applications remains unsatisfactory because of poor safety and low efficacy. Recently, nanomaterials (NMs) have emerged as a promising new strategy to strengthen both the efficacy and safety of ASCVD therapy. Thus, a comprehensive review of NMs used in ASCVD treatment will be useful. This paper presents an overview of the pathophysiological mechanisms of ASCVD and the multifunctional mechanisms of NM-based therapy, including antioxidative, anti-inflammation and antiapoptosis mechanisms. The technological improvements of NM delivery are summarized and the clinical transformations concerning the use of NMs to treat ASCVD are examined. Finally, this paper discusses the challenges and future perspectives of NMs in cardiac regeneration to provide insightful information for health professionals on the latest advancements in nanotechnologies for ASCVD treatment.

## Introduction

Atherosclerotic cardiovascular disease (ASCVD), the leading cause of disability and death worldwide, is an ischemic heart disease characterized by coronary artery stenosis or occlusion (Bejarano et al., 2018). The main treatments for ASCVD are operation and pharmacological intervention. At multiple stages of ASCVD development—including atherosclerosis (AS), stable ASCVD, unstable ASCVD, and acute cardiovascular events—pharmacological intervention is the main approach used to control blood lipids, blood pressure, and other risk factors associated with ASCVD. Most drugs used to treat ASCVD, however, are poorly soluble in physiological media and experience a considerable first-pass effect, resulting in reduced efficiency and some potential adverse effects. For instance, statins are the most commonly used drugs to relieve low density lipoprotein (LDL) cholesterol levels and limit the progression of atherosclerotic plaques, but they have the disadvantage of low oral bioavailability, and conventional doses can induce a variety of adverse reactions, such as muscle pain and diabetes, as well as rhabdomyolysis and liver function damage in severe cases (Schonewille et al., 2016). The difficulty of delivering sufficient concentrations of drugs to local lesions in a controlled manner is a major limitation of current treatments. Moreover, the currently available treatment strategies for AS only focus on the regulation of lipid metabolism while ignoring the reduction of inflammation. When ASCVD progresses to a severe stage, surgical treatment or thrombolytic therapy is the last option to boost blood perfusion and save dying cardiomyocytes (Cabac-Pogorevici et al., 2020). Because of the occurrence of myocardial infarction (MI), even if a patient receives adequate treatment, a large number of cardiomyocytes will have either died or suffered irreversible injury, and reperfusion injury will further aggravate myocardial injury. Therefore, even with timely treatment, many patients gradually progress to myocardial hypertrophy and heart failure, which is an important reason why ASCVD has a high fatality rate.

For a long time, scientists have been investigating the possible mechanisms related to the pathological process of ASCVD and regenerative repair, including oxidative stress, inflammation, calcium homeostasis disorders, lipid transport, apoptosis, and ventricular remodeling, aiming to discover innovative solutions to reverse the pathological process of AS and enhance the therapeutic effects of MI treatments (Zhang et al., 2017; Chan et al., 2018; Wu et al., 2018; Cabac-Pogorevici et al., 2020; Hu et al., 2020). A consistent gap exists, however, in the translation of these research advances into downstream clinical applications.

Nanomedicine is a multidisciplinary and cutting-edge field holding the potential to provide solutions for the prevention, diagnosis, and treatment of ASCVD (Hu et al., 2020). Generally, the nanomaterials (NMs) used in nanomedicine can be categorized as zero-dimensional [nanoparticles (NPs)], one-dimensional (nanotubes, nanofibers, nanorods, and nanowires), two-dimensional (nanofilms), or three-dimensional (bulk materials) NMs (Chen et al., 2021a)with diameters ranging from 1 to 100 nm. In the biomedical world, larger structures (1–1,000 nm) are also considered NMs (Bejarano et al., 2018; Lozano et al., 2018; Hu et al., 2020). As a result of their dimensions, NMs show unique physical and chemical properties, such as a high surface area-to-volume ratio, high reaction area, and high modifiable surface chemistry. Furthermore, because of their controllable shapes and sizes, NMs also exhibit properties superior to traditional therapeutics. In general, the pharmacokinetics and biodistributions of NMs are affected by their size, shape, surface, and mechanical properties. In detail, soft, spherical, or nonpositively charged NMs present longer circulation time and lower accumulation in the spleen and kidneys. Small-diameter NMs (diameter <5.5 nm) are more easily cleared by the kidneys, large-diameter NMs (diameter >150 nm) are more easily cleared by the mononuclear phagocyte system (MPS), and medium-diameter NMs (5.5–150 nm) are absorbed by tissue-resident macrophages, monocytes, and dendritic cells belonging to the MPS (Salvioni et al., 2019). Moreover, NMs can be customized to increase drug solubility, bioavailability, and specificity (Fernandes et al., 2020). Therefore, nanomedicines could modulate the biodistribution and target site accumulation of systemically administered chemotherapeutic drugs to meet the needs of different applications, thereby improving the curative effect while reducing the side effects and toxicity (van der Meel et al., 2019; Hu et al., 2020). In the past few decades, research progress in the fields of biotechnology, tissue engineering, and polymer science has accelerated the development of nanomedicine, leading to a number of applications being tested in preclinical trials with promising outcomes. More important, NM-based therapy is a promising strategy in the cardiovascular field with the potential to enhance the safety and efficacy of ASCVD treatment (Mura and Couvreur, 2012).

This review outlines the pathophysiological mechanisma of ASCVD and the mechanisms of NMs in treating AS and promoting heart tissue repair after MI, including antioxidant, anti-inflammatory, antiapoptosis, and antifibrosis mechanisms as well as mechanisms of improving lipid metabolism, maintaining calcium homeostasis, inhibiting extracellular matrix (ECM) degradation, and promoting angiogenesis. Furthermore, the improvements of NMs for cargo delivery in the cardiovascular field are summarized. Finally, the challenges in the application of NMs in the cardiovascular field, as well as the investigations of new promising NMs and strategies in this field, such as biomimetic NMs, are discussed. Taken together, this review provides researchers and clinicians with insightful information on the advancements in nanomedicine for ASCVD, with the purpose of promoting its application in downstream clinical applications.

## Pathophysiology of ASCVD and the Corresponding Nanomedicines for Medical Treatment

### Pathophysiology of ASCVD

**FIGURE 1:**
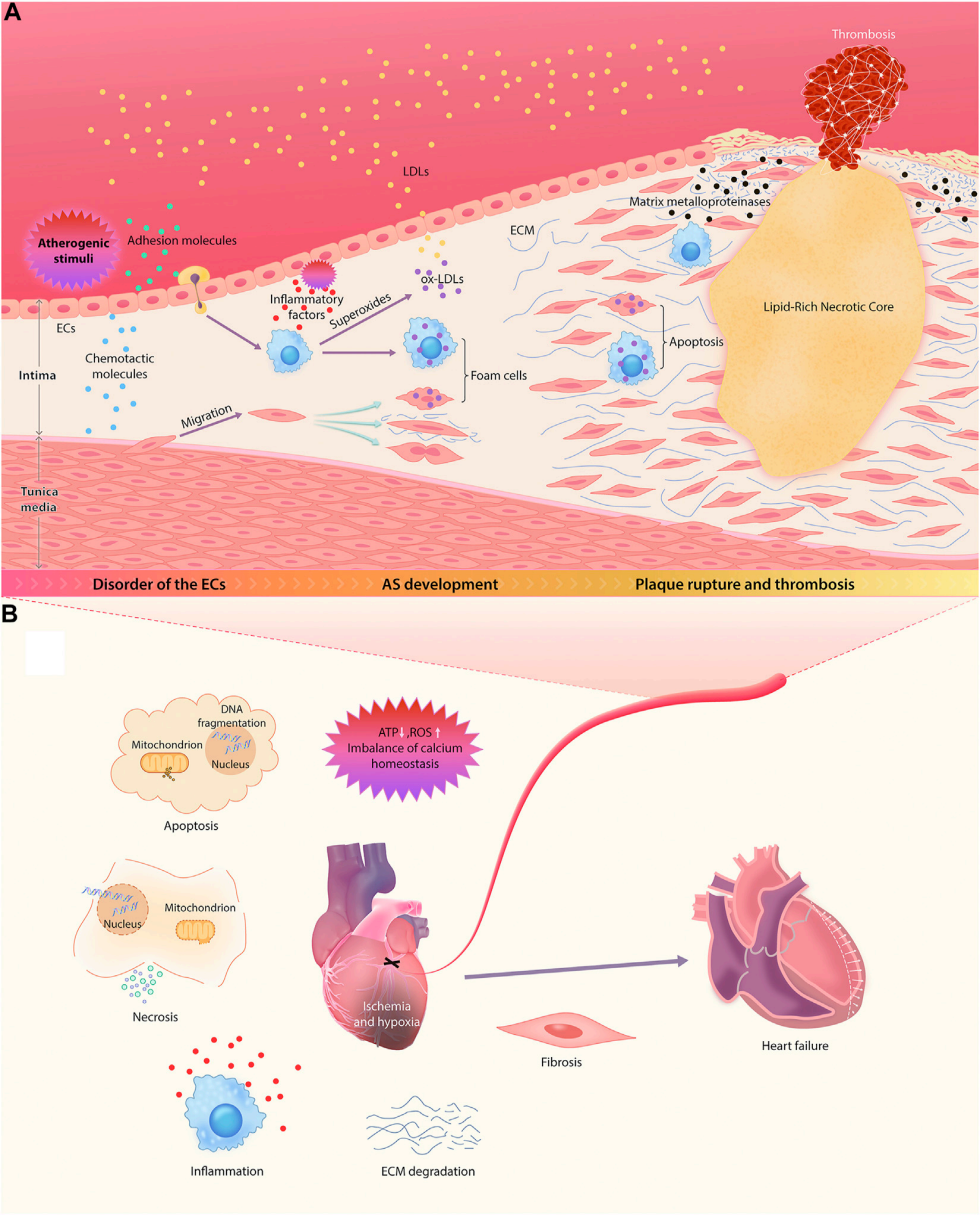
Pathophysiological mechanisms of ASCVD. **(A)** The sequential multistep process of AS, starting from endothelial dysfunction and ending in the rupture of unstable plaque. **(B)** The main mechanisms of the occurrence and development of myocardial injury and ventricular remodeling after the occlusion of diseased coronary artery.

This section outlines the complete pathophysiological process of ASCVD. For ease of understanding, a schematic diagram of the major features and events involved in this process is provided (Figure 1).

AS is an important pathological background for the occurrence and development of ASCVD. A large number of studies have shown that the formation of atherosclerotic plaque begins with endothelial dysfunction in large and midsize arteries (Bedenbender and Schmeck, 2020; Stromsnes et al., 2020; Carresi et al., 2021). Under normal circumstances, vascular endothelial cells (ECs) are vital for maintaining vascular integrity and permeability, and they resist the adhesion of circulating immune cells (Zhang et al., 2017).

However, atherogenic stimuli, including hypertension, hyperlipidemia, hyperglycemia, and inflammation, can upregulate the expression of EC adhesion molecules and chemotactic molecules (Gimbrone and García-Cardeña, 2016; Zhang et al., 2017), further enhancing the recruitment of monocytes and the abnormal migration and proliferation of vascular smooth muscle cells (VSMCs). This abolishes the monolayer structure of ECs, which increases the permeability of vascular walls and enhances the release of inflammatory factors, resulting in an increase in lipoprotein permeability (Ross and Glomset, 1973; Doran et al., 2008). Thereafter, cholesterol and other lipids gradually deposit in the walls of blood vessels.

Oxidized LDL (ox-LDL) and inflammation are the key contributors to the occurrence and development of AS. When LDL metabolism is aberrant, increased LDL in the blood permeates directly into vascular walls through the spaces between ECs. Infiltrating monocytes differentiate locally into macrophages, which can further release superoxides, hydroxyl radicals, and hydrogen peroxide to oxidize LDL and absorb a large amount of ox-LDL, resulting in the formation of foam cells. The continuous accumulation of foam cells induces fatty striations on the arterial wall, leading to the development of AS (Ye et al., 2019). Meanwhile, the recruited macrophagesalso can secrete a variety of mediators, such as interleukin-1 (IL-1), platelet–derived growth factor and transforming growth factor (TGF-α), which participate in the inflammation and immune responses and significantly accelerate the proliferation of VSMCs. The resultant abnormal proliferation of intimal VSMCs and the production and accumulation of ECM are the main contributors to local lumen stenosis and AS lesions (Doran et al., 2008; Poli et al., 2009). In addition, TGF-α and IL-1 produced by macrophages not only promote coagulation but also inhibit lipoprotein lipase (LPL) in adipocytes and the dissolution of lipids (Rajtar et al., 2006).

With the development of atherosclerotic plaque, the vascular intima gradually thickens. Once the oxygen diffusion threshold is exceeded, local hypoxia arises. Previous studies have shown that neovascularization is a compensatory mechanism for malnutrition and hypoxia (Hu et al., 2020). At the late stage of AS, however, neovascularization eventually becomes a channel for inflammatory cells (especially monocytes) to migrate to the lesion area, resulting in an increasing number of inflammatory cells in the intima (Hu et al., 2020). Inflammation is an important factor leading to plaque instability. Macrophages can induce apoptosis of smooth muscle cells and secrete various matrix metalloproteinases to degrade fibrous caps. This causes thinning of fibrous caps, making plaques more likely to rupture and bleed (Zhang et al., 2017).

In fact, the main cause of death in acute cardiac events is unstable plaque rupture and the subsequent pathological processes, namely thrombosis and MI. After MI, a microenvironment of continuous hypoxia leads to ATP deficiency, production of reactive oxygen species (ROS) and imbalance of calcium homeostasis, which triggers cell damage of different components of myocardium, including cardiomyocytes, ECs, fibroblasts, and stroma. This eventually results in the activation of apoptosis cascade and cell necrosis. Consequently, this process triggers a severe inflammatory response through an immune cascade, resulting in the activation of complements, the production of ROS, and the activation of inflammasomes (Cabac-Pogorevici et al., 2020). The subsequent release of a variety of proinflammatory mediators (such as cytokines and chemokines) induces the recruitment of inflammatory cells into the lesions of MI and enhances the inflammatory response after MI. By targeting the boundary area between infarcted tissue and healthy myocardial tissue, infiltrating leukocytes may induce additional cardiomyocyte death in this area, leading to the extension of ischemic and inferior myocardial remodeling beyond the original MI region (Ong et al., 2018). In addition, local oxidative stress injury and inflammation induce ECM degradation and promote tissue fibrosis, resulting in ventricular remodeling (Cabac-Pogorevici et al., 2020). Myocardial tissue is gradually replaced by scar tissue, and then cardiac function deteriorates progressively, eventually leading to the development of congestive heart failure.

### Nanomedicines for Medical Treatment

The main pathological mechanisms involved in the pathological process of ASCVD are oxidative stress, inflammation, and cell apoptosis, which contribute to the ventricular remodeling process after myocardium injury. In this section, the application of NMs treating ASCVD by acting on the noted mechanisms is discussed (Ohnishi et al., 2007; Pagliari et al., 2012; Soumya et al., 2014; Lewis et al., 2015; Park et al., 2015; Wang et al., 2015; Koppara et al., 2016; Navaei et al., 2016; Somasuntharam et al., 2016; Vrijsen et al., 2016; Beldman et al., 2017; Chen et al., 2017; Gallet et al., 2017; Hao et al., 2017; Li et al., 2017; Rufaihah et al., 2017; Chen et al., 2018; Hosoyama et al., 2018; Wu et al., 2018; Zhu et al., 2018; Zhang et al., 2019a; Zhang et al., 2019b; Peña et al., 2019; Sun et al., 2019; Dong et al., 2020; Gao et al., 2020; Lai et al., 2020; Tashakori-Miyanroudi et al., 2020; Zhang et al., 2021) (summarized in Table 1).

**TABLE 1:** Summary of the Nanomedicines used for ASCVD treatment targeting the mechanisms of ASCVD pathophysiology.

NPs	Therapeutic mechanisms	Biomaterial or cargo	Targeting moiety	Application	Animal model	Administration route	References
PEG-PPS	Antioxidant	Andrographolide	N.A	AS	Mouse AS model	Tail vein injection	Wu et al. (2018)
SGG	Antioxidant	Guar gum	N.A	Myocardial I/R injury	N.A	N.A	Soumya et al. (2014)
TDNs	Antioxidant	N.A	N.A	Myocardial I/R injury	N.A	N.A	Zhang et al. (2019a)
Mito-Fenozyme	Antioxidant	Tissue adhesive hydrogels	Mitochondria and TIM-2	Myocardial I/R injury	Mouse myocardial I/R injury model	Tail vein injection or cardiac patch	Zhang et al. (2021)
Nanoceria	Antioxidant	N.A	N.A	Stem cell therapy	N.A	N.A	Pagliari et al. (2012)
GO flakes	Antioxidant	N.A	N.A	Stem cell therapy for myocardial I/R injury	Rat myocardial I/R injury model	Intramyocardial injection	Park et al. (2015)
Fullerenol NPs	Antioxidant	Alginate hydrogel	N.A	Stem cell therapy for AMI	Rat MI model	Intramyocardial injection	Hao et al. (2017)
AM NPs	Anti-inflammation	N.A	Scavenger receptors	AS	Mouse AS model	Tail vein injection	Lewis et al. (2015)
HA NPs	Anti-inflammation	N.A	CD44, ICAM -1, LYVE-1, RHAMM and TLR-4	AS	Mouse and rabbit AS model	Tail vein injection	Beldman et al. (2017)
Dz-AuNPs	Anti-inflammation	N.A	TNF-α mRNA	MI	Rat MI model	Intramyocardial injection	Somasuntharam et al. (2016)
PP/PS@MIONs	Anti-inflammation	N.A	External magnetic field-induced targeting and PSR	MI	Rat MI model	Tail vein injection	Chen et al. (2017)
PzF nanothin layer	Anti-inflammation	N.A	N.A	CVD	Porcine coronary stent implantation model	Coronary stent implantation	Koppara et al. (2016)
iPSC-exosomes	Antiapoptosis	N.A	N.A	Myocardial I/R injury	Mouse myocardial I/R injury model	Intramyocardial injection	Wang et al. (2015)
iPSC-exosomes	Antiapoptosis	N.A	N.A	MI	Porcine MI model	Intramyocardial injection	Gao et al. (2020)
ADSC-exosomes	Antiapoptosis	N.A	N.A	Myocardial I/R injury	Mouse myocardial I/R injury model	Intramyocardial injection	Lai et al. (2020)
BMSC-exosomes	Antiapoptosis	N.A	N.A	Myocardial I/R injury	N.A	N.A	Sun et al. (2019)
Hypoxic BMSC-exosomes	Antiapoptosis	N.A	N.A	MI	Mouse MI model	Intramyocardial injection	Zhu et al. (2018)
Hypoxic BMSC-exosomes	Antiapoptosis	N.A	N.A	MI	Rat MI model	Intramyocardial injection	Zhang et al. (2019b)
Electrospinning cellulose nanofibers	Antiventricular remodeling	N.A	N.A	MI	Rat MI model	Cardiac patch	Chen et al. (2018)
BMSC-exosomes	Antifibrosis	N.A	N.A	MI	N.A	N.A	Ohnishi et al. (2007)
CDC-exosomes	Antiventricular remodeling	N.A	N.A	MI	Porcine myocardial I/R and MI model	Intramyocardial injection	Gallet et al. (2017)
CMPC- and MSC-exosomes	Promotion of regeneration	GR matrigel	N.A	Ischemic disease	N.A	Subcutaneous injection	Vrijsen et al. (2016)
PLGA nanofiber	Promotion of regeneration	N.A	N.A	MI	Rat MI model	Cardiac patch	Li et al. (2017)
GAG mimetic peptide nanofiber scaffold	Promotion of regeneration	N.A	N.A	MI	Rat MI model	Intramyocardial injection	Rufaihah et al. (2017)
AuNPs	Promotion of regeneration	Reverse thermal gel	N.A	MI	N.A	N.A	Peña et al. (2019)
Nano-gold-containing patche	Promotion of regeneration	Collagen	N.A	MI	Mouse MI model	Cardiac patch	Hosoyama et al. (2018)
GNR	Promotion of regeneration	GelMA	N.A	MI	N.A	N.A	Navaei et al. (2016)
Gold NPs	Promotion of regeneration	Extracellular matrix/silk proteins	N.A	MI	Rat MI model	Cardiac patch	Dong et al. (2020)
CNF	Promotion of regeneration	Collagen	N.A	MI	Rat MI model	Cardiac patch	Tashakori-Miyanroudi et al. (2020)

PEG-PPS, poly (ethylene glycol) and poly (propylene sulphide); N.A, not assessed; AS, atherosclerosis; SGG, selenium incorporated guar gum NPs; I/R, Ischemia/reperfusion injury; TDNs, tetrahedral DNA nanostructures; Mito-Fenozyme, mitochondria-targeted nanozymes; Nanoceria, cerium oxide NPs; MI, myocardial infarction; GO, graphene oxide; AM, sugar-based amphiphilic macromolecule; HA, hyaluronan; Dz-AuNPs, deoxyribozyme functionalized gold NPs; PP, poly (lactide)-polycarboxybetaine; PS, phosphatidylserine; MIONs, magnetic iron oxide nanocubes; PSR, phosphatidylserine receptors; PzF, Polyzene-F; iPSC, induced pluripotent stem cells; ADSC, adipose-derived stem cells; BMSC, bone marrow mesenchymal stem cells; CDC, cardiosphere-derived cells; CMPC, cardiomyocyte progenitor cells; MSC, marrow mesenchymal stem cells; PLGA, poly (D,L-lactic-co-glycolic acid); GAG, glycosaminoglycan; GNR, gold nanorod; GelMA, elatin methacrylate; CNF, carbon nanofibers.

#### Antioxidants

Oxidative stress contributes to the entire pathophysiological process of ASCVD, such as AS, MI, and ischemia/reperfusion (I/R) injury, because it aggravates disease progression by promoting inflammation (Chan et al., 2018; Wu et al., 2018; Cabac-Pogorevici et al., 2020; Hu et al., 2020). It also reduces the survival time and efficacy of transplanted cells used to treat MI. Although antioxidants are important for ASCVD management, they are rarely used in the current clinical environment. Encouragingly, the use of NMs with antioxidant capacity in ASCVD research is increasing year by year, and many such NMs have achieved significant curative effects.

For instance, using poly (ethylene glycol) and poly (propylene sulfide) block copolymer (PEG-PPS), Wu et al. constructed ROS-responsive polymer nanomicelles that can deliver andrographolide to the pathologic sites of AS to synchronously suppress oxidative stress and inflammation (Wu et al., 2018). After tail vein injection in mice, the micelles accumulate at atherosclerotic plaque, resolve in response to the oxidative microenvironment, and scavenge ROS, and then they release andrographolide to suppress inflammation. Accordingly, the AS level decreases obviously. Moreover, PEG-PPS reduces the systemic side effects of andrographolide and improves its water solubility. Another study reported that tetrahedral DNA nanostructures (TDNs) are synthesized using four specific DNAs. By activating the Akt/Nrf2 signaling pathway, TDNs inhibit oxidative damage by reducing the production of ROS and regulate the expression of apoptosis-related genes and proteins to inhibit cell apoptosis in myocardial injury induced by I/R (Zhang et al., 2019a). However, the design of TDNs does not consider targeting strategies, and the previous conclusions of *in vitro* studies have not been further verified in corresponding *in vivo* studies. Because of their high stability and large surface area, NMs with intrinsic enzyme-like activity (nanozymes) are considered to be one of the most promising alternatives to natural enzymes (Zhao et al., 2019). With ferritin-heavy-chain-based protein as the scaffold and a metal NP core as the active center, Zhang et al. designed TIM-2- and mitochondria-targeted nanozymes (Mito-Fenozymes) as superoxide scavengers that can target mitochondria in hypoxic cells by passing through multiple biological barriers. They used the Mito-Fenozymes in the treatment of cardiac I/R injury, marking the first time nanozymes were used for this purpose (Zhang et al., 2021). Through local release from transplanted cardiac patches or intravenous injection in cardiac I/R model mice, Mito-Fenozymes both effectively mitigate oxidative stress injury and further promote the regeneration of infarcted tissue, thereby improving the recovery of cardiac function.

Furthermore, Park et al. showed that, after intramyocardial injection, graphene oxide (GO) flakes can effectively scavenge ROS and thus improve the survival of MSCs in I/R myocardium. Then the enhanced secretion of reparative paracrine factors reduces apoptosis of cardiac tissue, enhances angiogenesis, and improves cardiac function (Park et al., 2015). Subsequently, Hao et al. further introduced fullerenol NPs into alginate hydrogel to design an injectable cell delivery vehicle that can effectively consume hydroxyl radicals and superoxide anions (Hao et al., 2017). Results suggested that the fullerenol/alginate hydrogel effectively alleviates the oxidative damage in the MI zone by activating the p38 and ERK pathways, increases the reservation and survival capacity of implanted brown adipose–derived stem cells (BADSCs), and further promotes angiogenesis and the recovery of cardiac function. In addition, the fullerenol/alginate hydrogel has no cytotoxic effects on BADSCs. The application of GO flakes and fullerenol NPs through thoracotomy, however, restricts clinical conversion.

#### Anti-Inflammation

Inflammation is the essential feature of AS, as it is closely related to the occurrence and development of AS and plaque instability (Zhang et al., 2017). Furthermore, excessive inflammation aggravates myocardial injury and enlarges the infarct size (Ong et al., 2018). Macrophages are the main cell type implicated in ASCVD development, and the M1/M2 classification is a convenient system for grouping different subpopulations of macrophages. Generally, M1 is dominant in disease progression through its proinflammatory effects, and M2 is dominant in disease regression through its anti-inflammatory effects (Shapouri Moghaddam et al., 2018). The lack of suitable anti-inflammatory therapy has become an important cause of the unsatisfactory treatment of ASCVD. Hence, numerous studies have directed their focus on anti-inflammatory NMs, and it is worth mentioning that macrophage polarization has important implications for the development of novel targeted therapies.

In research on AS, Lewis et al. synthesized sugar-based amphiphilic macromolecule NPs (AM NPs) by flash nanoprecipitation. The AM NPs competitively bind scavenger receptors (SRs) of macrophages, inhibit the uptake of ox-LDL and block the formation of foam cells and the subsequent inflammation (Lewis et al., 2015). *In vivo* studies have proved that AM NPs effectively suppress inflammation and the development of plaque in lesion areas. Subsequently, Beldman et al. designed hyaluronan (HA) NPs that selectively target plaque-associated macrophages and observed the significant anti-inflammation effects of HA NPs in mice plaques (Beldman et al., 2017). Furthermore, HA NPsalso can be used for positron emission tomography imaging of AS-associated inflammation, proving the great value of clinical translational application.

In research on acute myocardial infarction (AMI), Chen et al. designed a dual-targeting nanosystem (PP/PS@MIONs). Specifically, with phosphatidylserine (PS) targeting to PS receptors on macrophage surfaces and external magnetic field-induced targeting, PP/PS@MIONs accumulate in infarcted areas and accelerate the resolution of early inflammatory responses by promoting the polarization of macrophages to the reparative phenotype (M2) (Chen et al., 2017). Moreover, PP/PS@MIONs can realize the early magnetic resonance imaging of infarcted myocardial tissue. Despite the significant curative effects in AMI brought about by anti-inflammatory NMs, follow-up studies should be conducted to assess the long-term efficacy of such materials on cardiac remodeling and cardiac function.

Additionally, in-stent restenosis is an important risk factor for the poor prognosis of patients with ASCVD after percutaneous coronary intervention. Using nanomedicine to optimize the existing drug-eluting stent by inhibiting the inflammatory response on its inner surface may be an effective means to reduce the incidence of in-stent restenosis. Preclinical studies by Koppara et al. reported a new kind of coronary stent with a polymer NM as an important part, namely, COBRA Polyzene-F NanoCoated Coronary Stent (COBRA PzF NCS), which includes flat thin struts and a PzF nanoscale coating on its surface. PzF is a flexible fluorinated polymer. Through *in vivo* experiments, researchers have determined that the PzF nanoscale isolates stent struts from the vessel tissue and the circulating blood and suppresses the adherence of monocytes to repress inflammation and neointimal hyperplasia on the inner surface of stents placed in pig coronary arteries. Note that multiple clinical trials have verified the safety and effectiveness of the COBRA PzF NCS, which will be expand on in Section 4.

#### Antiapoptosis

The death of cardiomyocytes mainly occurs in the 24-h period following AMI, and apoptosis is an important form of cardiomyocyte death during MI (Cabac-Pogorevici et al., 2020). Many factors, such as hypoxia, oxidative stress, calcium homeostasis imbalance, and inflammation, can lead to apoptosis (Abbate et al., 2002; Zhou et al., 2020). Because of the slow self-renewal of cardiomyocytes, blocking the apoptotic pathway as early as possible to reduce cardiomyocyte death seems to be an important way to treat AMI. Exosomes are cell–derived nanoscale structures containing various biomolecules associated with intercellular communication and multiple physiological and pathological processes (Ali et al., 2020). Notably, exosomes from certain cells have outstanding performance in blocking apoptosis and promoting damage repair after MI (Xiong et al., 2021). However, because systemically administered exosomes cannot be effectively recruited to the MI myocardial tissue, intramyocardial injection, despite being invasive, remains the preferred method in most related studies. Interestingly, other studies have shown that the exosomal membrane can be chemically modified to improve the homing efficiency of intravenous exosomes (Armstrong et al., 2017). In this section, the antiapoptotic effect of exosomes will be highlighted.

As an acellular therapeutic option for myocardial injury, a variety of stem cell–derived exosomes can inhibit cardiomyocyte apoptosis after MI. Gao et al. determined that, by way of intramyocardial injection, exosomes secreted by induced pluripotent stem cells (iPSC) improve the recovery of MI model animals by reducing cardiomyocyte apoptosis with a low frequency of arrhythmogenic complications (Gao et al., 2020). *In vivo* and *in vitro* experiments conducted by Lai et al. indicated that I/R operation remarkably reduces miR-221/222 expression in cardiomyocytes and induces apoptosis and hypertrophy, whereas exosomes secreted by adipose–derived stem cells reverse all these effects. Subsequently, miR-221/222 have been proved to repress apoptosis and hypertrophy by suppressing the expression of the apoptosis-related protein PUMA and the hypertrophy-related protein ETS-1 in miR-221/222 knockout-mice (Lai et al., 2020). Furthermore, Sun et al. demonstrated that, by inhibiting Bax expression, miR-150-5p from bone marrow mesenchymal stem cell (MSC)–derived exosomes reduces the rate of cardiomyocyte apoptosis in MI mice (Sun et al., 2019).

Researchers also have attempted to strengthen the antiapoptotic effect by modifying natural exosomes. For instance, Zhu et al. suggested that, by upregulating miR-125b, hypoxic pretreatment enhances the antiapoptotic capability of bone MSC–derived exosomes in MI mouse cardiomyocytes (Zhu et al., 2018). Exosomes from miR-125b knockdown bone MSCs under hypoxic preconditioning, however, weaken the ability to repress cardiomyocyte apoptosis. Similarly, Zhang et al. increased the microRNA-24 of bone MSCderived exosomes by hypoxic preconditioning, thereby enhancing the inhibitory effect of bone MSCderived exosomes on cardiomyocyte apoptosis in MI rats (Zhang et al., 2019b).

#### Antiventricular Remodeling and the Promotion of Regeneration

During convalescence after MI, as a result of the slow renewal of myocardial cells, endogenous myocardial repair is insufficient to compensate for the cell loss caused by MI (Abbate et al., 2002). Therefore, ventricular remodeling and scar repair occur after MI, leading to progressive deterioration of cardiac function. Inhibition of ventricular remodeling is an important treatment during convalescence after MI, and enhancing the effective regeneration and repair of infarcted myocardial tissue can fundamentally reverse ventricular remodeling and improve heart function (Cabac-Pogorevici et al., 2020). Inhibiting myocardial remodeling and promoting myocardial regeneration and repair are both research hotspots in the application of NMs.

##### Antiventricular Remodeling

Using layer-by-layer coating technology, Chen et al. modified electrospun cellulose nanofibers with multiple layers of chitosan/silk fibroin to fabricate a nanofibrous patch that imitates the ECM of myocardium and ameliorates the post-MI microenvironment (Chen et al., 2018). When applied to infarcted regions in MI rats through thoracotomy, the nanofibrous patch distinctly curbs ventricular remodeling by restraining myocardial fibrosis. In addition, the cardio-protection of the nanofibrous patch can be further enhanced by carrying adipose MSCs. The mode of application being thoracotomy, however, restricts the clinical conversion of the nanomedicine. *In vitro* research from Ohnishi et al. revealed that MSC–derived exosomes inhibit fibrosis by promoting cardiac fibroblast proliferation and inhibiting the synthesis of types I and III collagen (Ohnishi et al., 2007). *In vivo* research from Gallet et al. suggested that, through intramyocardial injection rather than intracoronary infusion, exosomes from cardiosphere–derived cells significantly reduce collagen content and restrain cardiomyocyte hypertrophy to curb adverse ventricular remodeling in MI model pigs (Gallet et al., 2017).

##### Promotion of Regeneration

Numerous studies have shown that exosomes secreted by a variety of different cells can significantly promote angiogenesis after MI. For example, research from Vrijsen et al. has suggested that exosomes from both MSCs and cardiomyocyte progenitor cells, benefiting from the stimulating effect of EMMPRIN on ECs, have a significant role in promoting angiogenesis after MI (Vrijsen et al., 2016). Cardiomyocytes differentiated from human iPSCs are promising for myocardial regeneration, but most of the cells appear to be immature. This obviously impedes the application of human iPSC–derived cardiomyocytes in the repair of cardiac injury. Encouragingly, Li et al. cultured human iPSC–derived cardiomyocytes on low-thickness aligned nanofibers synthesized from biodegradable poly (D,L-lactic-co-glycolic acid) (PLGA) polymer and obtained myocardial tissue with more mature morphology and function, which is promising for clinical applications (Li et al., 2017). Notably, without adding any stem cells, exosomes, growth factors, or other biologically active molecules, Rufaihah et al. reported nanofiber scaffolds with synthetic glycosaminoglycan mimetic peptide as the active ingredient. When injected in the infarcted regions of MI model rats, the scaffolds significantly increase neovascularization in damaged myocardial tissue by promoting the expression of vascular endothelial growth factor A, and they improve the cardiac function of MI rats (Rufaihah et al., 2017). Compared with traditional thoracotomy, however, a minimally invasive surgery may be more conducive to the clinical translation of nanofiber scaffolds.

Furthermore, some other researchers have shown that electrically conductive NMs can be modified to mimic the myocardial ECM using tissue engineering techniques (Ashammakhi et al., 2019) and focus on the electrical and mechanical repair of infarcted myocardium by NMs (Ashtari et al., 2019). In such studies, metal NMs or carbon NMs are incorporated into biological materials, such as hydrogels, to improve electrical conductivity and mechanical stiffness. When used for MI therapy by local injection or patch, this scheme increases the uniform expression of connexin 43 (Navaei et al., 2016; Hosoyama et al., 2018; Peña et al., 2019), a cardiac specific marker, enhances the proliferation and expansion of cardiomyocytes (Dong et al., 2020; Tashakori-Miyanroudi et al., 2020), promotes angiogenesis (Hosoyama et al., 2018; Tashakori-Miyanroudi et al., 2020), and improves cell alignment and construction to repair contractile and conductive myocardial tissue irreversibly forfeited after MI.

## Nanomedicines for Drug Delivery

**FIGURE 2:**
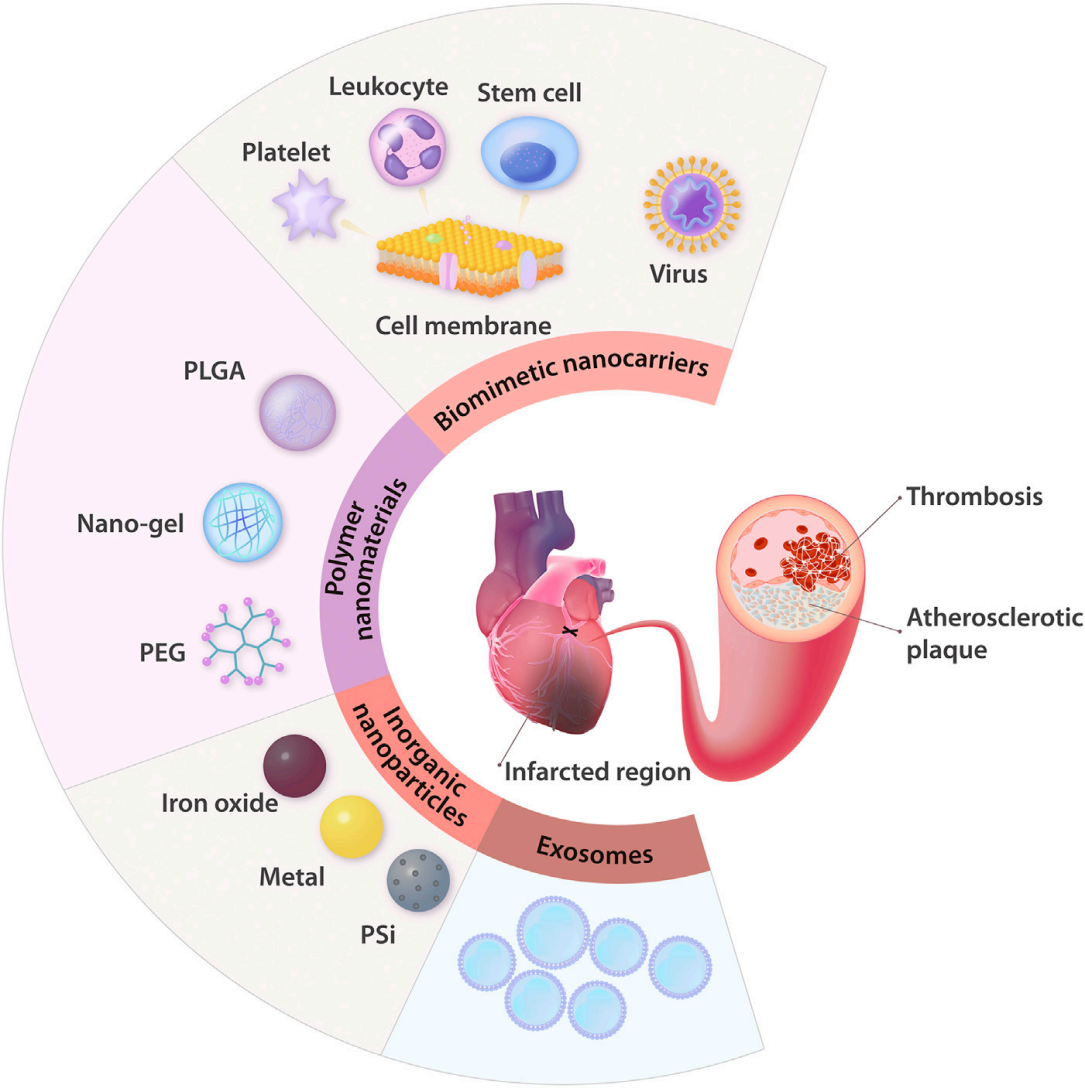
Summary of the common NMs for drug delivery applied in ASCVD treatment. In the therapeutic field of ASCVD, nanodelivery systems are mainly used to treat AS, thrombosis, and ischemic heart injury. The NMs most commonly applied in these systems are polymer NMs (e.g., PLGA, nano-gel, and PEG) and inorganic NPs (e.g., iron oxide NPs, other metal NPs, and PSi NPs). With the advantages of natural biological properties, exosomes, and cell membranes coated in NPs can serve as biomimetic nanocarriers, and this approach is increasingly being recognized as a brilliant strategy to treat ASCVD. PLGA: poly (D,L-lactic-co-glycolic acid); PEG: polyethylene glycol; PSi: porous silicon.

To ensure the maximal curative effect and low side effects, the nanodelivery systems should be able to stably encapsulate drugs and specifically target AS or MI lesions, and they also should be nontoxic (Cassani et al., 2020). Considering these ideal characteristics, NMs applied in nanodelivery systems for ASCVD are analyzed. At present, the most common NMs investigated for use as drug delivery systems in ASCVD research are polymer NMs, inorganic NPs, biomimetic nanocarriers, and exosomes (Figure 2).

### Polymer Nanomaterials

Because of their design flexibility and because they are easily functionalized to transport drugs to target lesions, polymers have been extensively investigated as drug-delivery NMs (Ekladious et al., 2019), and, of particular relevance to our review, one application is the treatment of ASCVD (Clemons et al., 2013; Katsuki et al., 2014; Fredman et al., 2015; Lee et al., 2015; Zhang et al., 2015; Sager et al., 2016; Bejerano et al., 2018; Mihalko et al., 2018; Nie et al., 2018; Xue et al., 2018; Lee et al., 2019; Nguyen et al., 2019; Wang et al., 2019; Yang et al., 2019), as summarized in Table 2. This section discusses recent advances in PLGA, polymeric nano-gels, and PEG to transport different classes of cargo.

**TABLE 2:** Summary of the polymer NPs for drug delivery in treating ASCVD.

Polymer carrier	Cargo/Therapeutic mechanisms	Targeting moiety	Application	Animal model	Administration route	References
RhB-PGMA	AID/Suppression of calcium overload	N.A	MI	Guinea pig I/R model	Coronary perfusion	Clemons et al. (2013)
PLGA	Pitavastatin/Anti-inflammation	N.A	AS	Mouse plaque destabilization and rupture model	Tail vein injection	Katsuki et al. (2014)
PLGA-b-PEG	GW3965/Anti-inflammation	N.A	AS	Ldlr−/− mouse AS model	Tail vein injection	Zhang et al. (2015)
HA	N.A	Stabilin-2 or CD44	AS	ApoE-deficient mouse AS model	Tail vein injection	Lee et al. (2015)
PLGA-PEG	Collagen IV-binding peptide/Anti-inflammation	Ac2-26	AS	Ldlr−/− mouse AS model	Intravenous injection	Fredman et al. (2015)
PEI600	SiRNA/Anti-inflammation	Icam1 and Icam2, Vcam1, and E- and P-selectins	MI	ApoE−/−mouse I/R model	Intravenous injection	Sager et al. (2016)
Poly (N-isopropylacrylamide) nano-gel	tPA and Y-27632/Thrombolysis and antifibrosis	Fibrin	MI	Rat I/R model	Intracoronary injection	Mihalko et al. (2018)
PEG-DGL	AMO-1/Antiapoptosis	AT_1_R	MI	Mouse MI model	Tail vein injection	Xue et al. (2018)
PGEA	miRNA-499 and pVEGF/Antiapoptosis and proangiogenesis	N.A	MI	Mouse MI model	Eye canthus intravenous injection	Nie et al. (2018)
HA	miRNA-21/Anti-inflammation	Macrophage	MI	Mouse MI model	Tail vein injection	Bejerano et al. (2018)
PLGA	GSH/Antioxidant	CD44	MI	N.A	N.A	Lee et al. (2019)
DSPE-PEG	miRNA-199a-3p/Proangiogenesis	CPPs	Myocardial I/R injury	Rat I/R model	Intramyocardial injection	Yang et al. (2019)
TPP	miRNA-33/Regulation of cholesterol efflux	Macrophage	AS	N.A	N.A	Nguyen et al. (2019)
PDA	TIIA/Antioxidant and anti-inflammation	Targeting moiety	MI	Rat MI model	Intramyocardial injection	Wang et al. (2019)

RhB-PGMA, rhodamine B-polyglycidal methacrylate; AID, a peptide derived against the alpha-interacting domain; N.A, not assessed; MI, myocardial infarction; PLGA, poly (D,L-lactic-co-glycolic acid); AS, atherosclerosis; PLGA-b-PEG, poly(lactide-co-glycolide)-b-poly(ethylene glycol); GW3965, a liver X receptor agonist; HA, hyaluronic acid; Icam, intercellular cell adhesion molecules; Vcam 1, vascular cell adhesion molecule 1; PEI600, polyethyleneimine with a molecular weight of 600; tPA, tissue plasminogen activator; Y-27632, a small molecule cell contractility inhibitor; PEG-DGL, poly(ethylene glycol)-dendrigraft poly-l-lysine; AMO-1, specific microRNA-1 inhibitor; AT_1_R, angiotensin II type 1 receptor; PGEA, ethanolamine-modified poly(glycidyl methacrylate); GSH, heparin and glutathione; I/R, Ischemia/reperfusion injury; DSPE-PEG, distearate phosphatidylethanolamine-polyethylene glycol; CPPs, cell-penetrating peptides; TPP, tripolyphosphate; PDA, polydopamine; TIIA, tanshinone IIA.

Over the past 15 years, research on drug delivery systems using PLGA NPs in treating ASCVD has soared exponentially. Numerous PLGA NPs for the delivery of growth factors, bioactive peptides, agonists or inhibitors of receptors, and various small-molecule drugs have been developed and even used in clinical practice. Some studies have explored the use of such NPs for the treatment of AS, AMI, and myocardial I/R injury (Simón-Yarza et al., 2013; Katsuki et al., 2014; Zhang et al., 2015; Ma et al., 2018; Lee et al., 2019; Yokoyama et al., 2019). For instance, to alleviate the activation of immune activation after MI, Fujiwara et al. loaded TAK242, an inhibitor of Toll-like receptor 4, in PLGA NPs for the treatment of myocardial I/R injury through intravenous injection (Fujiwara et al., 2019). The results demonstrated that TAK242 transportation prevents Ly-6C high monocytes from being recruited to injured heart tissue, thus avoiding the ensuing overactive local inflammation.

Polymeric nano-gels possessing all the necessary characteristics for drug delivery are easy to synthesize, and their size can be controlled as well. Therefore, they have been applied in multiple fields, including drug delivery, bioengineering, diagnostics, and sensing (Chacko et al., 2012). The emergence of nano-gels has greatly enriched and promoted the application of polymer NMs in treating ASCVD (Chen et al., 2013; Grimaudo et al., 2019; Prajnamitra et al., 2019). For example, Tang et al. devised thermosensitive nano-gels encapsulating therapeutic cells to treat MI through intramyocardial injection and showed that the nano-gels protect transplanted cells from attack by immune cells and also provide an environment conducive to their survival, indicating great value and potential for clinical use (Tang et al., 2017). Mihalko et al. cleverly synthesized a double-layer nano-gel with a core-shell structure to individually control the release of two distinct molecules, tissue plasminogen activator and cell contractility inhibitor, which simultaneously endow the dual-delivery system with the functions of targeting thrombolysis and mitigating I/R injury (Mihalko et al., 2018).

This paper specifically focuses on PEG, a highly water-soluble, biocompatible, nonconductive, and flexible polymer widely used in drug delivery. When combined with PEG, therapeutic or other functional drugs for treating ASCVD, such as proteins, nucleic acids, or other NMs, can facilitate immunoevasion and be isolated from various enzymes and renal filtration *in vivo* through steric repulsion to enhance the blood circulation time (Paulis et al., 2012; Fredman et al., 2015; Lewis et al., 2015; Zhang et al., 2015; Shao et al., 2017; Ma et al., 2018; Xue et al., 2018; Banik et al., 2020). Puerarin (PUE) is made from pueraria tuberosa, and its main chemical component is PUE flavone, which can enhance myocardial contractility and inhibit cardiac hypertrophy and myocardial apoptosis through the regulation of mitochondrial function (Chen et al., 2014; Chen et al., 2021b). However, very little free PUE can reach the mitochondria of ischemic cardiomyocytes. To solve this problem, Wen et al. devised a mitochondrial targeting PEG-PE modified with triphenylphosphonium (TPP) cations to target PUE to mitochondria (Li et al., 2019). When administered *via* tail intravenous injection in Balb/c mice, the drug delivery system significantly enhances the blood circulation time and increases the accumulation and retention of PUE in the ischemic myocardium to reduce cardiomyocyte apoptosis.

### Inorganic Nanoparticles

Common inorganic NPs, which mainly include comprising metal NPs, and silicon NPs, can be easily and extensively fabricated (Labatut and Mattheolabakis, 2018) and have been widely studied in basic research associated with ASCVD.

Because of the biological properties of metallic NPs, they have been used for treating myocardial I/R injury, AMI, and heart failure (Miragoli et al., 2018; Chang et al., 2019; Chen et al., 2019; Lee et al., 2020). With biocompatibility and low toxicity, iron oxide NPs are the most common metal NM used in the study of ASCVD. Moreover, the excellent paramagnetic properties also endow iron oxide NPs with the ability to directionally convey drugs, exosomes (Lee et al., 2020), and a variety of stem cells (Ottersbach et al., 2018; Zhang et al., 2019c) to the heart with magnetic field guidance. For example, Liu et al. designed an NP composed of a Fe_3_O_4_ core and a shell of silica modified by PEG and conjugated to antibodies to target injured cardiomyocytes and exosomes. They demonstrated that it mediates targeted aggregation of exosomes from circulation to infarcted heart tissue to play a therapeutic role with magnetic field guidance (Liu et al., 2020). This design causes the scattered exosomes in the autologous blood circulation to enter the MI area for treatment, successfully avoiding the potential immunogenicity of exogenous exosomes and other risks. The source of circulating exosomes is complex, however, and the different biologically active components they carry may have opposite effects on the repair of MI injury. Therefore, knowing how to identify and carry therapeutic exosomes in the blood circulation to the local area may limit the clinical translation of the innovative design.

Silicon NPs are another common inorganic NM attracting increasing attention. Porous silicon (PSi) shares the characteristics of unique chemical surface and biodegradability, which are conducive to controlling drug release. Ferreira et al. synthesized atrial natriuretic peptide-loaded PSi NPs, which target specific natriuretic peptide receptors in cardiomyocytes and cardiac fibroblasts. By intramyocardial injection, the engineered NPs accumulate in the heart of MI model rats, especially in the endocardial layer of the ischemic region, and significantly attenuate hypertrophic signaling (Ferreira et al., 2017). Another study reported a cell-penetrating peptide-conjugated PSi loaded with Wnt3a protein, which aggregates in MSCs and increases the activity of antioxidative stress (Qi et al., 2019). Furthermore, PSi can load genetic drugs for cardiac repair (Yao et al., 2020).

### Biomimetic Nanocarriers

Nanocarrier designs that utilize biomimetic strategies, such as cell membrane coating nanotechnology, are increasingly recognized as brilliant methods to generate nanocarriers with increased blood circulation time, reduced clearance by reticular MPS, and the capability to target diseased tissue (Fang et al., 2018).

The use of cell membrane coatings on drug delivery NPs is an inspiring step forward. With adhesion antigens and the ability of immunomodulation, the platelet membrane has been used in many delivery systems of nanomedicines to cure cardiovascular diseases. For example, inspired by the inherent activities of platelets in hemostasis and thrombosis, Yao et al. designed polymer NPs camouflaged by platelet membranes for recombinant tissue plasminogen activator (rt-PA) delivery to thrombus sites and determined that the biomimetic nanomedicine, in comparison with free rt-PA, has better efficacy and a lower bleeding risk *in vivo* experiments (Xu et al., 2020). Song et al. devised PLGA NPs camouflaged in platelet membranes for the delivery of rapamycin (RAP-PNP) (Song et al., 2019a). Because of its innate affinity to AS plaques, after being injected in AS model mice, RAP-PNP automatically homes to AS sites, markedly curbs AS progression, and stabilizes plaques. Moreover, the PNP delivery system simultaneously reduces the systemic side effects of RAP.

Leukocytes, naturally coexisting in harmony with the immune system, prevent uptake and target inflamed tissues through interactions with cellular membranes (Fang et al., 2018). In detail, using lymphocyte function-associated antigen one in their membrane, leukocytes can trigger the clustering of intercellular adhesion molecule one on activated ECs so that they can bind to inflamed endothelium and traverse across layers of endothelium to reach diseased tissue (Palomba et al., 2016). Therefore, the leukocyte membrane is naturally suitable for nanodelivery designs for cardiovascular diseases. By encapsulating NPs with leukocyte membranes, the resulting biomimetic nanomedicine perfectly replicates the characteristics of leukocytes, preferentially adheres to inflamed endothelium, and accelerates the transendothelial delivery of loaded drugs (Parodi et al., 2013). Because the functional components of the leukocyte membrane include various proteins on its surface, Molinaro et al. integrated membrane proteins extracted from leukocytes into lipid NPs and synthesized a novel proteolipid vesicle. The subsequent results of *in vivo* experiments indicated that proteolipid vesicles could preferentially adhere to inflamed vasculature and effectively transport dexamethasone into inflammation sites to suppress inflammation (Molinaro et al., 2016). Encouragingly, the leukocyte membrane coating nanotechnology has been successfully used for the treatment of myocardial I/R injury in recent preclinical studies. For instance, Zhang et al. showed that, by modifying extracellular vesicles from MSCs with monocyte mimics through membrane fusion, the vesicles present the recruitment feature of monocytes to I/R lesions, overcoming the disadvantage of poor homing efficiency and significantly improving the curative effect on myocardial I/R model mice (Zhang et al., 2020).

Moreover, different types of stem cells are also used as raw materials for biomimetic nanotechnology. Yao et al. reported that an MSC membrane coating endows mesoporous silica NP loaded with miR-21 with improved immune escape as well as the ability to target and cure cardiomyocytes in MI lesions (Yao et al., 2020). To further improve the homing rate and application value of MSCs, Lee et al. co-cultured MSCs with iron oxide NPs (IONPs) and then, through cell extrusion using microporous filters, obtained IONP-containing nanovesicles, which exhibit similar function and size to MSC–derived exosomes and are magnetic (Lee et al., 2020). Subsequently, through intramyocardial injection and magnetic guidance, the nanovesicles successfully promote early transfer from the inflammation promotion phase to the reparative phase, significantly alleviate apoptosis and fibrosis, and enhance the healing of cardiac function. In addition, Pitek et al. indicated that one plant viral NPs can also target thrombi and exhibited their capability for streptokinase delivery in thrombosis model animals (Pitek et al., 2018).

### Exosomes

Cell–derived exosomes have the inherent characteristics of physicochemical stability, excellent biocompatibility and the capability to interconnect with target cells through signaling, fusion, and delivery, making them particularly suitable to be pharmaceutical carriers.

Among various nanodelivery systems, exosomes are the main effectors of miRNA carriage (Cassani et al., 2020). Transfection of target genes into specific cells through genetic engineering technology to obtain exosomes rich in genes is the most common form of exosomal drug-carrying treatment of ASCVD. For example, Song et al. transfected MiR21 expression plasmids into human embryonic kidney cell line, a commonly used immortalized cell line for exosome production, to generate exosomes rich in miRNA-21 (miR21-EVS), and the results of *in vivo* and *in vitro* experiments showed that miR21-EVS remarkably restrains the degradation of miRNA-21 by RNase, significantly represses the apoptosis of different kinds of heart cells by reducing programmed cell death protein four expression, and improves cardiac function (Song et al., 2019b). By transfecting MSCs with miR-150-5p antagomir and upregulating stromal cell-derived factor-1 (SDF1) using SDF1 plasmid respectively, Wu et al. and Gong et al. obtained exosomes rich in those components. Both interventions significantly strengthen the inhibitory effect of MSC-exosomes on cardiomyocyte apoptosis and improve cardiac function in MI model mice (Gong et al., 2019; Wu et al., 2021). Ni et al. transduced human umbilical cord MSCs with lentiviral-TIMP2 to generate exosomes rich in TIMP2 and reported that the engineered exosomes mitigate post-MI remodeling (Ni et al., 2019).

Through electroporation or transfection, exogenous material can be directly introduced into exosomes. For instance, Wu et al. introduced hexyl-5aminolevulinate hydrochloride (HAL) into exosomes from M2 macrophages through electroporation to produce HAL@M2 exosomes (Wu et al., 2020). After tail intravenous injection, the HAL@M2 exosomes, by means of the chemokine receptor on the M2 exosome membrane, adhere to inflammatory ECs, migrate across the ECs and ultimately congest in the AS site. Subsequently, owing to the ingestion by inflammatory cells, anti-inflammatory cytokines, and HAL in HAL@M2 exosomes are released and jointly suppress inflammation. Notably, as a product of HAL metabolism, protoporphyrin IX could be used for further fluorescence imaging to trace AS. Youn et al. reported that exosomes from cardiac progenitor cells transfected by miR-322 *via* electroporation distinctly strengthen the signaling axis of Nox2-ROS to mediate proangiogenesis and enhance the curative efficacy on MI model mice (Youn et al., 2019). Another study transfected exosomes from bone MSCs with miR-19a/19b mimics and demonstrated that the engineered exosomes reduce the apoptosis rate of cardiac HL-1 cells suffering from hypoxia injury (Wang et al., 2020).

In addition, non-gene cargo also can be introduced into exosomes to obtain additional functions. Huang et al. pretreated MSCs with atorvastatin and obtained atorvastatin-containing exosomes (Huang et al., 2020). Through intramyocardial injection, these exosomes increase the expression of vascular endothelial growth factor, miR-675, and intercellular adhesion molecule-1 by upregulating lncRNA H19 to promote the function of ECs and angiogenesis and enhance the curative effect on AMI rats.

## Clinical Translation of Nanomedicines in ASCVD

Although NMs have been popular in preclinical research on ASCVD treatments over the past several decades, their clinical translation has lagged in comparison (Kanthi et al., 2020; Pala et al., 2021). A search on the clinicaltrial.gov website in May 2021 identified only 10 clinical trials on the application of NMs for ASCVD-related nanomedicine (Table 3). This section presents some recent examples.

**TABLE 3:** Summary of clinical trials related to NMs (ClinicalTrials.gov).

Study title	Application (s)	Interventions	First posted	NCT number	Stage	Status
Clinical Performance of Nano Plus Sirolimus-Eluting Stents in Patients With Coronary artery disease	CVD	Device: Nano	October 10, 2016	NCT02929030	Not Applicable	Recruiting
Safety and Efficacy of the Combo Bio-engineered Sirolimus-eluting Stent Versus the Nano Polymer-free Sirolimus-eluting Stent in the Treatment of Patients With de Novo Stenotic Lesions	CVD	Device: OrbusNeich Combo stent™	September 4, 2015	NCT02542007	Not Applicable	Active, not recruiting
		Device: sirolimus-eluting stent system				
Efficacy and Safety of Nano + Polymer-free Sirolimus-Eluting Stent: A Optical Coherent Tomography Study	CVD	Device: Nano + DES	August 19, 2013	NCT01925027	Phase 4	Unknown
TReAtmeNt of Small Coronary Vessels: MagicTouch Sirolimus Coated Balloon	CVD	Device: SCB	April 12, 2019	NCT03913832	Not Applicable	Recruiting
		Device: paclitaxel releasing coronary balloon catheter				
Plasmonic Nanophotothermal Therapy of AS	CVD and AS	Procedure: Transplantation of NPs	January 5, 2011	NCT01270139	Not Applicable	Completed Has Results
		Procedure: Transplantation of iron-bearing NPs				
		Device: Stenting				
Plasmonic Photothermal and Stem Cell Therapy of AS Versus Stenting	CVD and AS	Other: Stenting and micro-infusion of NP	September 19, 2011	NCT01436123	Phase 1	Terminated
		Device: Implantation of everolimus-eluting stent				
Randomized Trial of COBRA PzF Stenting to Reduce Duration of Triple Therapy	CVD	Device: COBRA PzF	November 3, 2015	NCT02594501	Not Applicable	Active, not recruiting
		Device: Drug Eluting Stent				
Safety and Effectiveness Evaluation of COBRA PzF Coronary Stent System: A Post Marketing Observational Registry	CVD	Device: COBRA PzF	April 6, 2017	NCT03103620	Not Applicable	Completed
The PzF Shield Trial	CVD	Device: COBRA PzF	August 20, 2013	NCT01925794	Not Applicable	Unknown
COBRA SHIELD OCT Study	CVD	Drug: Aspirin	August 25, 2014	NCT02224235	Not Applicable	Terminated
		Device: Resolute Integrity DES				
		Device: COBRA PzF				
		Drug: DAPT				

CVD, cardiovascular disease; DES, Drug-eluting stent; SCB, sirolimus drug coated balloon; AS, atherosclerosis; PzF, polyzene-F; DAPT, dual antiplatelet therapy.

In 2017, on the basis of preclinical research mentioned earlier in this review, Cutlip et al. reported the results of a multicenter, prospective, single-arm, nonrandomized clinical trial concerning COBRA PzF NCS. Strictly screened cardiovascular disease (CVD) patients (n = 115) received COBRA PzF NCS placement through a minimally invasive cardiac surgery and dual antiplatelet therapy (DAPT) for 30 days and experienced a 9-month follow-up. Compared with the historical results from contemporary studies that included bare-metal stent cohorts, COBRA PzF NCS met the performance goals with an excellent safety profile and no stent thrombosis (Cutlip et al., 2017). The clinical research protocol requires 30 days for DAPT, but patients with a very high risk of bleeding need a shorter DAPT duration. In 2020, Maillard et al. determined that in the context of patients with high bleeding risk who received mono antiplatelet therapy (Maillard et al., 2020a), COBRA PzF NCS is still safe and effective. This holds true in daily clinical practice for a high-bleeding-risk population with multiple comorbidities and complex situations (Maillard et al., 2020b). Subsequently, a more rigorous clinical trial (NCT02594501) (Colleran et al., 2021) further verified these conclusions, implying that COBRA PzF NCS is expected to become a potential alternative to current devices in daily clinical practice.

Two other studies, published in 2015 and 2017, jointly reported the results of the NANOM-FIM trial (NCT01270139) concerning silica-gold NPs and plasmonic photothermal therapy for the management of atheroprotective plaques in CVD patients. In detail, through a mini-surgery, the researchers implanted a bioengineered on-artery patch containing silica–gold NPs onto the diseased artery. Then they executed percutaneous or transcutaneous intercostal near-infrared laser irradiation at 821 nm to activate the NPs 1 week later (Kharlamov et al., 2015; Kharlamov et al., 2017). The 1-year follow-up analysis showed that patients who underwent the therapy had decreased necrotic cores and atheroma volumes and better rates of mortality, and the 5-year follow-up analysis further demonstrated the high safety and effectiveness of the nanotechnology. Thismodality of treatment, however, is complicated and may cause safety hazards, which is not conducive to its promotion and application in clinical settings.

## Limitations and Prospects

Although functionalized NMs can be used as smart drug delivery systems to improve curative effects and lessen side effects, nanomedicine still faces some challenges. After entering the blood but before they reach their targets, 30–99% of NPs of all sizes, shapes and chemical compositions will accumulate and sequester in the liver because of the phagocytosis of Kupffer cells, leading to serious off-target effects (Zhang et al., 2016). Obviously, absorption by the MPS is one of the main obstacles that almost all NPs must overcome (Nie, 2010). Additionally, some NPs may penetrate and persist in multiple organs for a long time, such as the liver, spleen, and kidneys (Boey and Ho, 2020). A few studies have shown that NMs, such as metal NPs, carbon nanostructures, and silica NPs, can produce severe side effects (Pan et al., 2009; Mei et al., 2012; Yin et al., 2013; Bostan et al., 2016; Boey and Ho, 2020; Hussain et al., 2020). These side effects are related to NP concentration, composition, agglomeration, modification, and route of administration. For instance, the toxicity of metal NMs is largely determined by their size (Boey and Ho, 2020). The toxicity of NMs is at least partially mediated by the inflammatory response of neutrophils and macrophages, and the ROS produced by these inflammatory responses further mediate damage to the body (Foulkes et al., 2020). Many studies have shown that surface modification to reduce absorption by MPS and to add specific targeting mechanisms can reduce the dose and toxicity and promote preferential targeted accumulation (Wu et al., 2015; Carnovale et al., 2016; Xu et al., 2016). Thus far, introducing PEG onto the surface of NPs has been the gold standard to decrease MPS clearance and increase blood circulation time (Suk et al., 2016). The use of PEG, however, cannot completely prevent MPS clearance (Immordino et al., 2006), and over multiple administrations, the existence of antibodies of PEG could lessen performance (Parodi et al., 2013). At present, other stealth polymers, such as poly (2-oxazoline)and poly (zwitterions), have shown better performance than PEG, but further verification is needed (Fam et al., 2020). Additionally, because of the demand for more powerful surface functionalities, the strategies of targeting ligand conjugation are becoming increasingly difficult, particularly for extensive manufacturing. It is true that the addition of targeting molecules would increase production costs, but the efficacy of the therapy could be significantly improved (Kedmi et al., 2018). Characterized by cell membrane coating nanotechnology, biomimetic nanomedicine offers the advantages of good availability, a wide range of options, natural immune escape ability, inherent targeting, and easy modification. For example, cell membrane guised or CD47 functionalized NPs have active biological components for *in vivo* antiphagocytic effects, which significantly enhance their potential in drug delivery. Moreover, further modifications with ligands, such as antibodies, peptides, enzymes, or proteins, would endow biomimetic platforms with better synergistic performance (Fam et al., 2020). Hence, it may be one of the best strategies to overcome the limitations of NMs, which has been preliminarily proven by some recent studies (Molinaro et al., 2016; Fang et al., 2018; Pitek et al., 2018; Xu et al., 2020).

## Conclusion

In spite of great progress in clinical practice and promotion of healthier lifestyles, ASCVD is and most likely will remain the leading cause of mortality worldwide in the coming decades (Bejarano et al., 2018). Remarkable advances in the fields of biotechnology, tissue engineering, bionics, and polymer sciences have enabled the development of NMs with additional characteristics, such as controlled release, immune evasion, and long blood circulation time, which has boosted the development of NMs for medical applications. Furthermore, from early arterial inflammation to ventricular remodeling, a comprehensive understanding of ASCVD pathogenesis could facilitate the discovery and understanding of new molecular markers and their functions, further driving the application of nanomedicine toward new and more effective targets for the treatment of ASCVD (Cassani et al., 2020). Herein, this work has reviewed a wide range of nanotechnology-based medical treatments and strategies of drug delivery in the field of ASCVD. Our hope is that this paper provides researchers and clinicians with insightful information on the advancements in nanomedicine for treating ASCVD, further unlocking the potential for downstream clinical applications. Although there are some obstacles and challenges in the development of NMs and only a small number of NMs have powerful evidence sustaining their clinical use, NMs have the potential to become better and more comprehensive alternatives to the current treatment options for ASCVD in the future.
